# Understanding Heterogeneity and Tolerance of Dual *Candida albicans–Staphylococcus aureus* Biofilms to Cold Atmospheric Plasma and Antimicrobial Combinations

**DOI:** 10.1111/apm.70119

**Published:** 2025-12-29

**Authors:** Ross M. Duncan, Thomas P. Thompson, Gordon Ramage, Ryan Kean, Laura A. McClenaghan, Yiwei Tian, Michael M. Tunney, Brendan F. Gilmore

**Affiliations:** ^1^ School of Pharmacy Queen's University Belfast Belfast UK; ^2^ Department of Nursing and Community Health Glasgow Caledonian University Glasgow UK; ^3^ Department of Biological and Biomedical Sciences Glasgow Caledonian University Glasgow UK

**Keywords:** amphotericin B, biofilms, cold atmospheric plasma, interkingdom, vancomycin

## Abstract

Interkingdom polymicrobial biofilms of *Staphylococcus aureus* and *Candida albicans* increase pathogenicity and complicate treatment strategies, contributing to greater morbidity and mortality. These infections are typically investigated using the *C. albicans* strain SC5314, despite its uncertain clinical relevance. Here, we evaluate how different *C. albicans* (high, intermediate, low biofilm formers) and *S. aureus* strains influence biofilm tolerance to both conventional antimicrobials and cold atmospheric plasma (CAP). CAP is an emerging technology that has been shown, including in our prior work, to disrupt biofilm biomass via reactive oxygen and nitrogen species, positioning it as a promising adjunct to antimicrobial therapy. We determined how strain variation influenced biofilm structure and tolerance to vancomycin, amphotericin B and CAP. Interkingdom biofilm architecture was primarily influenced by the *C. albicans* strain, particularly its ability to form hyphae. Despite this, all strains conferred protection to *S. aureus* against vancomycin. CAP eradicated *S. aureus* biofilms within 120 s, but this effect was lost in dual‐species biofilms. However, CAP pretreatment enhanced the efficacy of both antimicrobials in interkingdom biofilms. These findings highlight the role of hyphal morphology in vancomycin tolerance and support further clinical evaluation of CAP as a biofilm‐targeting adjunct.

## 1 Introduction

Interkingdom dual‐species biofilms formed by *Candida albicans* and *Staphylococcus aureus* pose a significant clinical challenge [1], often exacerbating infection severity and complicating treatment [2]. These organisms are frequently co‐isolated in bloodstream infections [3], the sputum of CF patients [4] and a growing number of osteomyelitis cases [5], yet the factors driving their interactions and treatment responsiveness remain poorly understood.

Synergistic interactions within these biofilms promote both pathogenicity and antimicrobial tolerance. Hyphal association facilitates *S. aureus* mucosal penetration and systemic spread [6], while shifting *C. albicans* towards a more virulent state, upregulating key biofilm and virulence genes such as *HSP90* and *ACE2* [7]. The architecture of *C. albicans* biofilms has also been shown to provide protection for *S. aureus* against a range of antimicrobials including vancomycin [8, 9] and miconazole [10], whereas *C. albicans* does not gain additional protection against antifungals [8, 11].

Despite extensive research on *C. albicans* – *S. aureus* biofilms, most studies rely on a single type strain of *C. albicans* , SC5314, or its derivatives (DAY 185, CAI4) [6, 7, 8, 9, 10, 11, 12, 13, 14, 15, 16]. However, a recent analysis of 224 *C. albicans* genomes revealed that only 33% of isolates contain the dominant ROB1946^S^ allele of the *Rob1* transcription factor associated with both increased filamentation and biofilm formation in SC5314 [17], raising concerns about its clinical representativeness.

Given the limited efficacy of antimicrobials against dual‐species biofilms, alternative strategies such as cold atmospheric plasma (CAP) are being investigated. CAP generates reactive oxygen and reactive nitrogen species (RONS) and has been extensively used in water purification [18] and has demonstrated antimicrobial activity across bacteria and fungi [19, 20, 21], including mono‐species biofilms [22, 23, 24, 25, 26, 27]. While the complete eradication of *S. aureus* biofilms using CAP has been described [28], *C. albicans* biofilms exhibit a greater tolerance [29, 30]. Furthermore, there is strong evidence to suggest low exposure of CAP to host cells is not only well tolerated but can aid in both cell proliferation and tissue regeneration, ultimately making it a desirable technology for use in wound healing [31, 32, 33].

In this study, we characterised interkingdom biofilms using *C. albicans* strains representing high (HBF), intermediate (IBF) and low (LBF) biofilm‐forming phenotypes, alongside *S. aureus* USA300 (a clinically relevant strain), Newman (a weak biofilm former) and a high‐biomass clinical isolate. We assessed *S. aureus* tolerance to vancomycin and *C. albicans* tolerance to amphotericin B within dual‐species biofilms, with and without CAP treatment. By linking strain composition, interspecies interactions and treatment responses, this study provides insight into CAP as a biofilm‐targeting adjunct for drug‐tolerant polymicrobial infections.

## 2 Materials and Methods

### 2.1 Strains and Cultures

The bacterial strain *S. aureus* BAA‐1717 was obtained from ATCC. *C. albicans* SC5314, NCYC 610 and ATCC 18804, as well as *S. aureus* Newman and NUI0017, were already present in the QUB strain collection. *C. albicans* strains were grown on Sabouraud dextrose agar (SDA [Oxoid Ltd., UK]) at 30°C for 48 h and maintained at 4°C. Tryptone Soya Agar (TSA [Oxoid Ltd., UK]) was used to grow *S. aureus* strains at 37°C for 24 h and maintained at 4°C.

### 2.2 Biofilm Formation of Yeast in Different Growth Media

*C. albicans* was resuspended in Sabouraud dextrose broth (SDB [Oxoid Ltd., UK]) and incubated at 30°C with shaking at 200 rpm overnight. Cultures were then centrifuged at 4000 × g for 15 min, rinsed twice in Phosphate Buffer Saline (PBS [Thermo Fisher Scientific, Paisley, UK]), resuspended and enumerated on a haemocytometer (Hawksley, UK). Cells were diluted in either RPMI‐1640 (Sigma Aldrich, UK) or SDB to reach a final concentration of 1.0 × 10^6^ cells/mL. 100 μL of each standardised culture was added to wells of a flat‐bottom, 96‐well plate (Thermo Fisher Scientific, UK) and incubated at 37°C for 24 h in a static incubator.

### 2.3 Growth of Mono‐ and Dual‐Species Biofilms

Enumeration of *C. albicans* was carried out as previously described. Overnight cultures of *S. aureus* in Tryptone Soya Broth (TSB [Oxoid Ltd., UK]) were centrifuged at 4000 × g for 15 min and washed twice with PBS before being adjusted to an OD_550_ = 0.15–0.18, which is equal to (1.0 ± 0.2) × 10^8^ CFU/mL, confirmed using Miles and Misra counts. *S. aureus* was then diluted in RPMI‐1640 to a final concentration of 1.0 × 10^6^ CFU/mL. Dual species biofilms were formed using a culture consisting of a 1:1 ratio of *C. albicans* : *S. aureus* both at a final concentration of 1.0 × 10^6^ cells/mL. 100 μL of mono and dual cultures were used to inoculate wells of a 96‐well plate. Plates for both mono‐ and dual‐species biofilm formation were incubated for 24 h at 37°C in a static incubator.

### 2.4 Quantification of Biofilm Biomass Using Crystal Violet Assay

Biomass quantification of fungal, bacterial and dual‐species biofilms was carried out as described by Sherry and co‐workers [34]. Briefly, 24 h biofilms were rinsed with PBS before being exposed to 0.05% w/v crystal violet (Sigma Aldrich, UK) for 5 min. Biofilms were then rinsed 3 times with PBS before destaining with 100% ethanol (Sigma Aldrich, UK). Ethanol was transferred to a fresh 96‐well plate before the absorbance was read at 570 nm on a FLUOstar Omega plate reader (BMG Labtech, Ortenberg, Germany).

### 2.5 Microscopic Analysis of Yeast Cell Morphology in Biofilms

*C. albicans* biofilms grown in 96‐well plates were rinsed with PBS before 100 μL of Calcofluor white solution (Sigma Aldrich, UK) was added, and plates were incubated in the dark for 10 min. The Calcofluor white was removed, and wells were rinsed twice with PBS. Wells were imaged on an EVOS M5000 fluorescent microscope using a DAPI filter (Ex./Em. 357/447 nm). Calcofluor white has a similar optimum Ex. (380 nm) and Em. (475 nm) spectrum to DAPI, making the filter suitable for imaging.

### 2.6 Quantification of Biofilm‐Associated Cells

Biofilms were washed with 100 μL PBS and then scraped using individual P200 tips to dislodge cells before adding 100 μL fresh PBS. Plates were sonicated for 20 min in a Branson 3510 sonic bath to uniformly disperse aggregated cells before enumeration. Cell suspensions in each well were serially diluted and plated using the Miles and Misra method [35] as follows: SDA was used for mono‐species *C. albicans* biofilms, and SDA + 10 μg/mL of chloramphenicol (Sigma Aldrich, UK) was used for the selection of *C. albicans* in dual‐species biofilms. TSA was used for enumeration of *S. aureus* in mono‐species biofilms, and Mannitol Salt Agar (MSA[Millipore, UK]) was used for the selection of *S. aureus* in dual‐species biofilms. SDA plates were incubated at 30°C for 48 h, while TSA and MSA plates were incubated at 37°C for 24 h.

### 2.7 Determining Minimum Biofilm Inhibition Concentration (MBIC) of Vancomycin and Amphotericin B Against *C. albicans* and *S. aureus* Biofilms

Mono‐species biofilms grown for 24 h were washed with 100 μL of PBS before 100 μL of either 8, 16, 32 or 64 μg/mL of vancomycin hydrochloride (Sigma Aldrich, UK) or 0.5, 1, 2, 4 or 8 μg/mL of amphotericin B (Cayman Chemical Company, USA) were added to wells. Plates were then incubated for a further 24 h at 37°C without shaking. 5 μL of 10X Alamar blue (Thermo Fisher Scientific, UK) was added directly to the antimicrobial‐containing medium within each well for a final volume of 105 μL (0.5X Alamar Blue), including positive and negative controls. Plates were incubated for the optimum times of each organism based on previous work by Peeters et al. [36] (30 min for *S. aureus* biofilms and 60 min for *C. albicans* biofilms) before fluorescence was quantified on a plate reader at an excitation of 544 nm and an emission of 590 nm. MBIC was determined as the lowest concentration of antimicrobial agent that resulted in a 50% reduction in biofilm metabolic activity compared to positive controls, as described elsewhere [37]. MBIC values of vancomycin and amphotericin B against mono‐species biofilms were used to determine the antimicrobial concentrations to be used against interkingdom biofilms (1×, 2× and 4× MBIC).

### 2.8 Survival of Interkingdom Biofilms After Treatment With Vancomycin or Amphotericin B

Interkingdom biofilms grown for 24 h were rinsed with 100 μL of PBS before 100 μL of 1×, 2× or 4× the MBIC concentration of either vancomycin or amphotericin B was added. Plates were incubated statically at 37°C for 24 h. The number of surviving *S. aureus* cells was determined for vancomycin treatments, and the number of surviving *C. albicans* cells for amphotericin B treatments was determined using the Miles and Misra method as described above.

### 2.9 Cold Atmospheric Plasma Treatments

**FIGURE 1:**
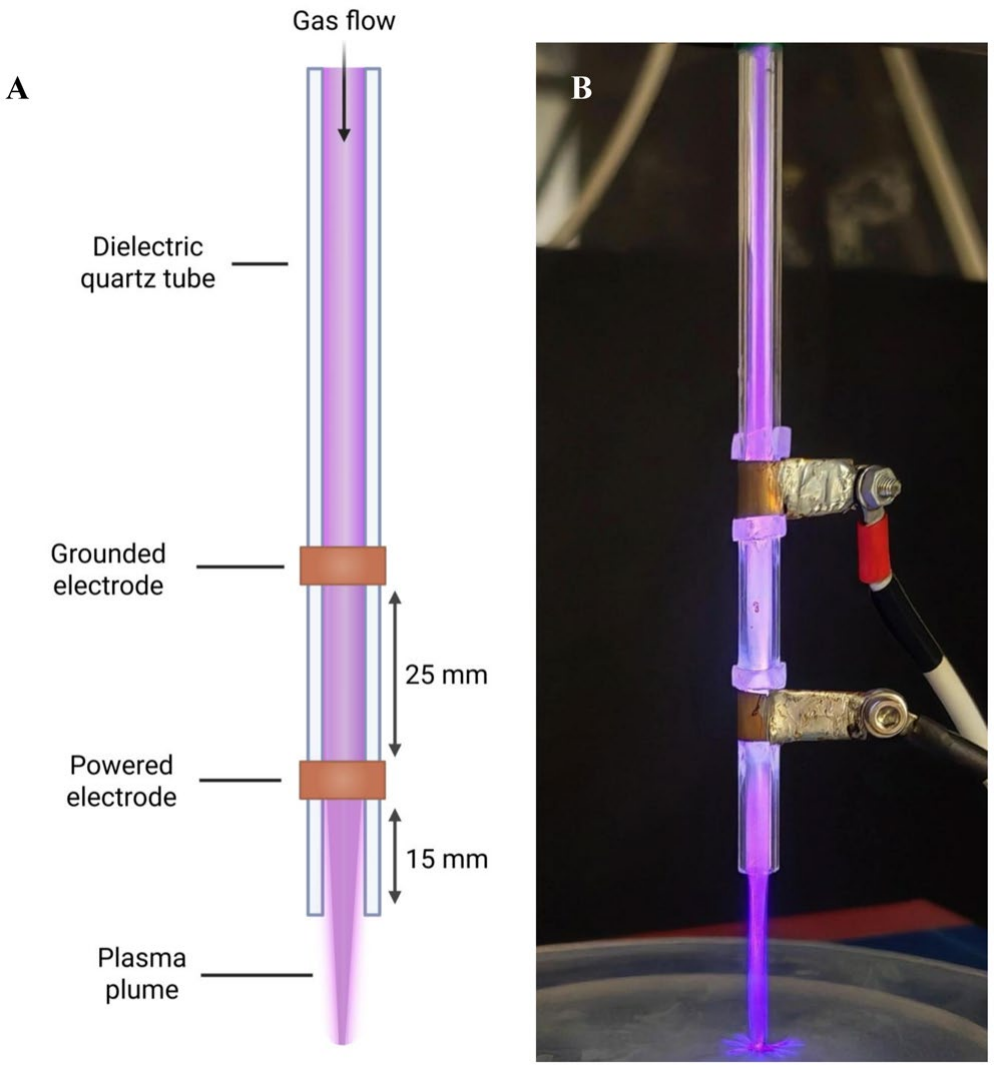
Cold atmospheric plasma jet design and operation. Schematic (A) and image (B) of the CAP jet in operation. Created in BioRender. https://BioRender.com/cg0y9oo

The in‐house kHz Jet, consisting of a dielectric quartz tube (6 mm outer diameter and 4 mm inner diameter) with a copper power electrode (15 mm from the exit of the quartz tube) and a copper grounded electrode (25 mm up from the power electrode), was operated at 6 kV with a repetition frequency of 20 kHz as described previously [26, 38, 39, 40, 41], with the adjustment of a 99.99% Helium feed gas at 2 standard litres per minute (Figure 1). The exit of the jet was set to 5 mm from the top of the well of the 96‐well plate for all treatments. Biofilms were washed with PBS, and all surface liquid was removed prior to CAP treatments. Biofilms were then treated with CAP, before either vancomycin, amphotericin B or both were added at different concentrations (16, 32 and 64 μg/mL and 0.5, 1 and 2 μg/mL, respectively) within RPMI‐1640. Plates were then incubated for 24 h at 37°C in a static incubator, and cell survival was evaluated using the Miles and Misra method.

### 2.10 Reactive Oxygen and Nitrogen Species Measurements

CAP treatments were carried out as described above with 100 μL of water in the bottom of wells in a 96‐well plate. To quantify hydrogen peroxide (H_2_O_2_), nitrate (NO^−^_3_) and nitrite (NO^−^_2_), the Amplex Red Hydrogen Peroxide/Peroxidase Assay Kit (ThermoFisher Scientific, UK), the Nitrate Spectraquant Assay Kit (Sigma Aldrich, UK) and the Griess Reagent Kit (Sigma–Aldrich, UK) were used, respectively. All kits were used as per the manufacturer's manuals and as described previously within our group [27].

### 2.11 Statistical Analysis

All statistical analysis and graph production were performed using GraphPad Prism (Version 10.4.2; GraphPad Software Inc., La Jolla, CA). Principal Component Analysis (PCA) was performed using the prcomp() function and visualised with ggplot2, ggrepel and dplyr in RStudio.

## 3 Results

### 3.1 Classification of *C. albicans* Biofilm‐Forming Ability

**FIGURE 2:**
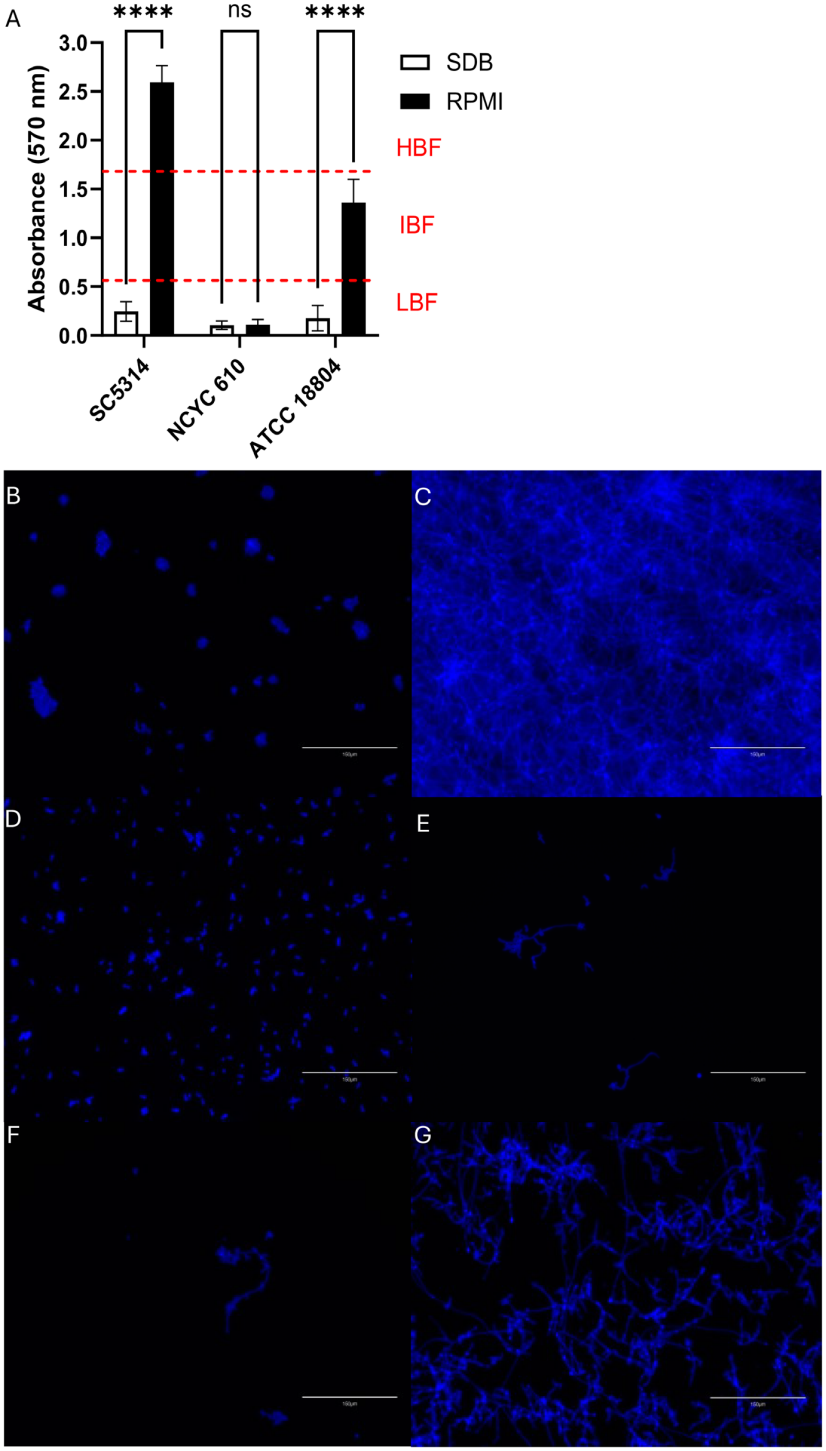
Total biomass of *C. albicans* strains grown in different growth media. *C. albicans* SC5314, NCYC 610 and ATCC 18804 biofilms were grown in SDB or RPMI for 24 h, stained with crystal violet and quantified by absorbance (570 nm) (A). *n* = 18. Significance is represented by ****p < 0.0001 determined by two‐way ANOVA test, followed by Šídák's multiple comparisons test. HBF, IBF and LBF sections are based on data from Sherry et al. [34]. Biofilms were stained with Calcofluor white and imaged on an EVOS m5000 microscope using a DAPI filter. Each panel represents a mono‐species biofilm, and the growth media used. SC5314 in SDB (B) and RPMI (C), NCYC 610 in SDB (D) and RPMI (E) and ATCC 18804 in SDB (F) and RPMI (G).

We aimed to characterise strain‐specific differences in *C. albicans* biofilms using total biomass quantification and fluorescent imaging. Figure 1 shows the variation in biofilm biomass and cell morphology among the three *C. albicans* strains across two media. The well‐characterised *C. albicans* SC5314 showed minimal biomass grown in SDB, with much greater biofilm biomass when grown in RPMI (Figure 2A). In SDB, SC5314 remained predominantly in the yeast form, with only a small proportion attaching to the well surface and clustering together (Figure 1B). In contrast, RPMI induced extensive hyphae formation, and a thick, multi‐layered meshwork covered the entirety of the well (Figure 2C). This was the most robust biofilm‐forming strain, with an average absorbance of 2.59 at 570 nm. *C. albicans* NCYC 610 exhibited no significant difference in total biomass between RPMI and SDB (*p* = 0.999), with an average absorbance of approximately 0.1 OD570 in both media. Microscopy confirmed a complete absence of hyphae or pseudohyphae formation in both conditions. Like SC5314, ATCC 18804 exhibited low total biomass (OD570 = 0.18) in SDB, with predominantly yeast cell morphology and minimal hyphae formation. However, when grown in RPMI, the total biomass of ATCC 18804 biofilms was significantly greater (OD570 = 1.36) compared to when it was grown in SDB and a much larger surface coverage by pseudohyphae (Figure 2F,G). Using the biofilm cut‐offs for clinical *C. albicans* strains originally suggested by Sherry et al. [34], we have classified SC5314 as a HBF, NCYC 610 as a LBF and ATCC 18804 as an IBF.

### 3.2 Interkingdom Biofilm Characteristics Are Influenced by the *C. albicans* Strain Present

To examine the influence of *C. albicans* strain variability, we compared interkingdom biofilms formed with high, intermediate and low biofilm‐forming strains (SC5314, ATCC 18804 and NCYC 610, respectively), alongside three *S. aureus* strains with distinct biofilm phenotypes. These included USA300, Newman and a clinical high‐biomass isolate (NUI0017).

**FIGURE 3:**
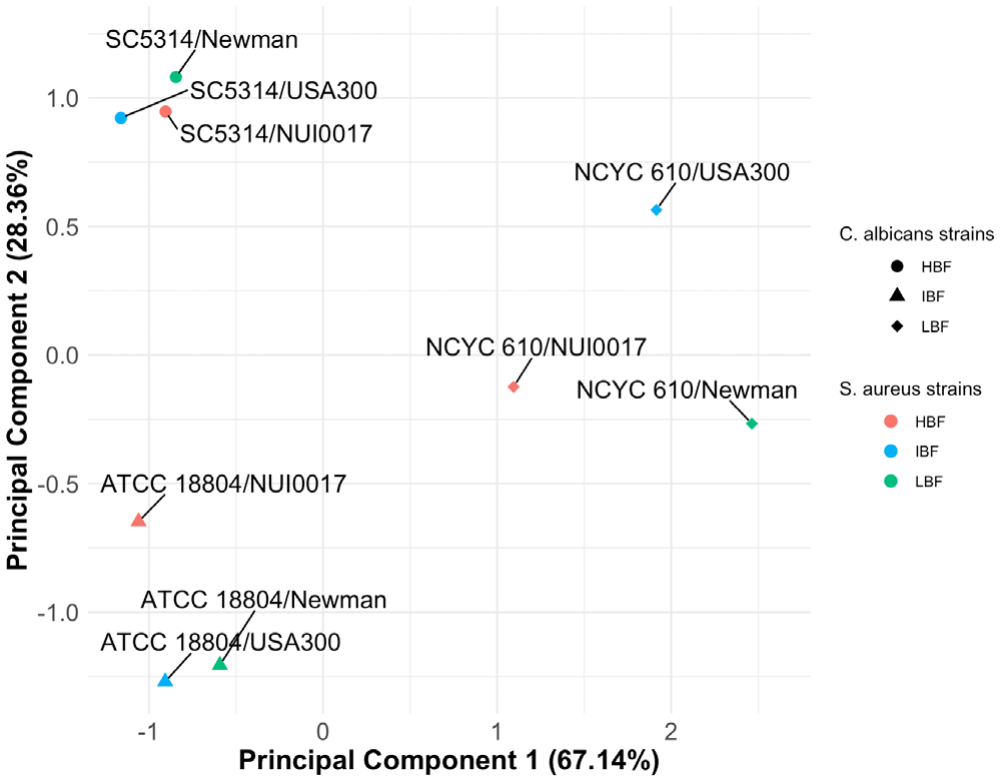
PCA plot of interkingdom biofilm relatedness based on total biomass, cell counts of *S. aureus* and *C. albicans* cells after 24 h. Biomass was determined using the CV assay, and *S. aureus* and *C. albicans* cell numbers were calculated using the Miles and Misra method.

Using total biomass and respective cell counts of *S. aureus* and *C. albicans* (Figures S1–S3), we assessed the relatedness of interkingdom biofilms via PCA (Figure 3). In the PCA, proximity reflects the similarity of physical properties. Interkingdom biofilms sharing the same *C. albicans* strain cluster more tightly than those sharing the same *S. aureus* strain, indicating the fungal strain exerts greater influence on overall structure. For example, biofilms containing SC5314 grouped tightly regardless of the *S. aureus* partner, whereas those containing NUI0017 were more dispersed depending on the *C. albicans* strain. While ATCC 18804 clusters closely with *S. aureus* USA300 and Newman, it diverged when paired with the HBF *S. aureus* strain NUI0017, suggesting that NUI0017 exerts a stronger influence over ATCC 18804 biofilm formation compared to SC5314. In contrast, NCYC 610 biofilms exhibited the greatest variation, indicating a stronger influencer of the *S. aureus* strain. Overall, these findings suggest that interkingdom biofilm characteristics are primarily dictated by the *C. albicans* strain, while *S. aureus* plays a secondary role. The primary factor influencing *C. albicans* ' role in interkingdom biofilms is its ability to form hyphae, which supports higher biomass accumulation and increased *S. aureus* attachment and proliferation.

### 3.3 Antimicrobial Tolerance of Mono‐ and Dual‐Species Interkingdom Biofilms

**FIGURE 4:**
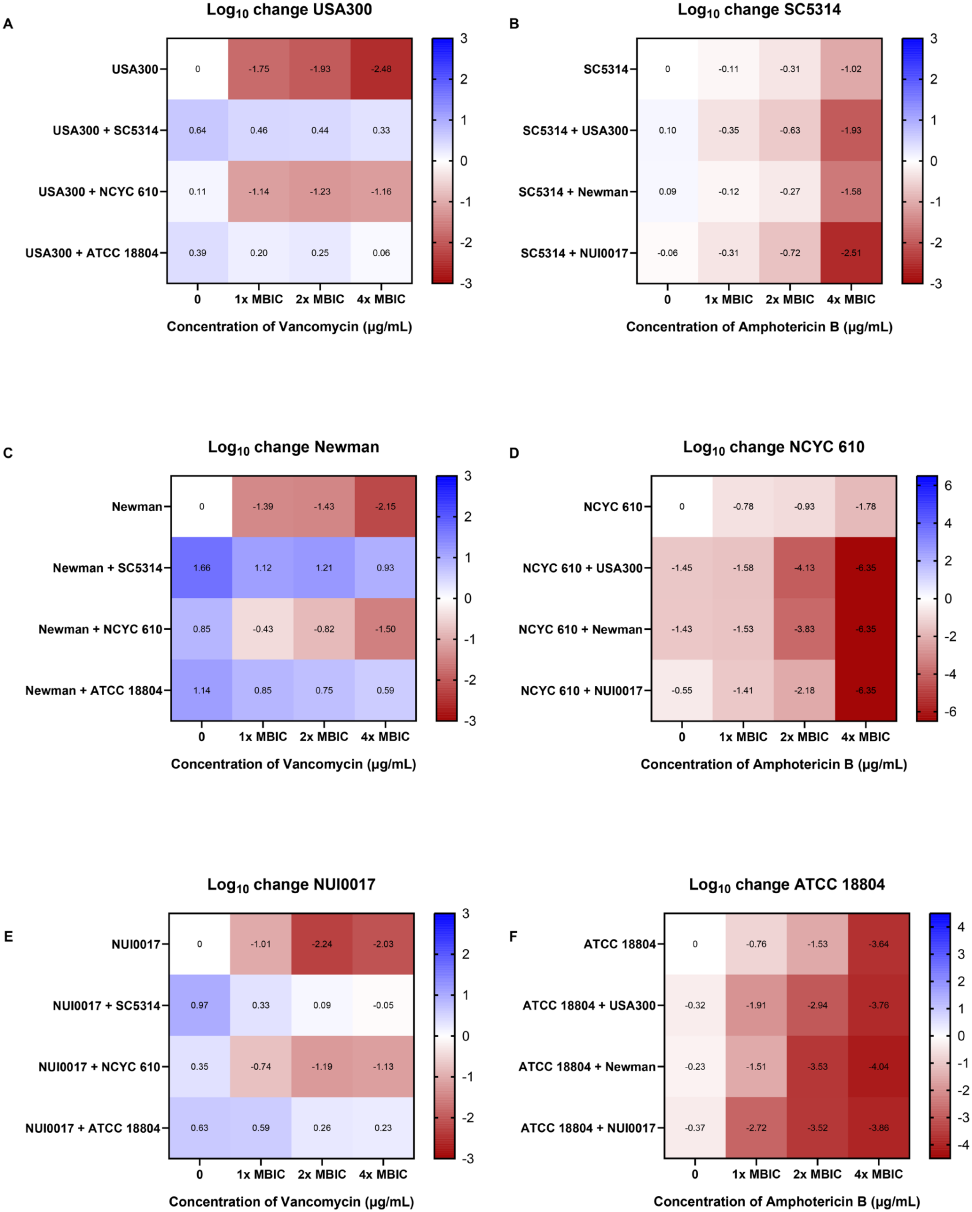
Log change in number of *S. aureus* and *C. albicans* cells in mono‐ and dual‐species biofilms after antimicrobial treatment. For *S. aureus* cell counts (A, C and E), mono‐ and dual species biofilms were treated with vancomycin concentrations at 1× MBIC, 2× MBIC or 4× MBIC. For *C. albicans* cell counts (B, D and F), mono‐ and dual‐species biofilms were treated with amphotericin B at 1× MBIC, 2× or 4× MBIC. Log changes in either *S. aureus* cell counts (A, C and E) or *C. albicans* cell counts (B, D and F) were calculated based off the control mono‐species biofilm of each strain with no antimicrobial challenge (top left corner) and represented on the scale bar on the right of each graph. Red = decrease in log CFU/mL, Blue = increase in CFU/mL.

The MBIC of vancomycin and amphotericin B for all *C. albicans* and *S. aureus* strains was determined as a baseline reference (Table 1). MBIC values for interkingdom biofilms could not be determined, as the unaffected non‐target species likely masked the antimicrobial challenge, dominating biofilm metabolism. This insensitivity likely allowed the resistant organism to dominate the biofilm, resulting in little to no observed decrease in metabolic activity. To address this limitation, cell survival was used as an indicator of antimicrobial tolerance. To quantify tolerance, colony recovery of each species was quantified following 1×, 2× and 4× the MBIC treatment (Figure 4).

**TABLE 1:** MBIC of amphotericin B and vancomycin against *S. aureus* and *C. albicans* strains used in this study.

Organism	Vancomycin MBIC (μg/mL)	Amphotericin B MBIC (μg/mL)
*S. aureus* BAA 1717 (USA300)	8	> 64
*S. aureus* NCTC 10833 (Newman)	16	> 64
*S. aureus* NUI0007	8	> 64
*C. albicans* SC5314	> 256	0.5
*C. albicans* NCYC 610	> 256	0.5
*C. albicans* ATCC 18804	> 256	2

*Note:* MBIC of vancomycin and Amphotericin B was determined as the first concentration where a 50% reduction in metabolic activity was observed in mono‐species biofilm using the Alamar blue assay.

For all three *S. aureus* strains, co‐culture SC5314 or ATCC 18804 significantly increased vancomycin tolerance compared to mono‐species biofilms (*p* < 0.0001). With SC5314, recoveries increased by 2.81‐, 3.08‐ and 1.98‐log for USA300, Newman and NUI0017, respectively, treated with 4× the MBIC of vancomycin. With ATCC 18804, recovery increased by 2.54‐, 2.74‐ and 2.26‐log in USA300, Newman and NUI0017 (*p* < 0.0001), respectively (Figure 4A,C,E).

When grown alongside *C. albicans* NCYC 610, all three *S. aureus* strains also exhibited increased tolerance to vancomycin with increased *S. aureus* cell recoveries of 1.32‐, 0.65‐ and 0.9‐log compared to the mono‐species biofilms of USA300, Newman and NUI0017 mono‐species biofilms (*p* < 0.0001), respectively. Though it should be noted that the protective effect provided by *C. albicans* NCYC 610 was not as substantial as that provided by the HBF and IBF.

*C. albicans* generally became more susceptible to amphotericin B when co‐cultured with *S. aureus* . When treated at 4× the MBIC of amphotericin B, SC5314 biofilms exhibited an additional 0.91 −log (*p* < 0.0001), 0.56‐log (*p* = 0.003) and 1.49 −log (*p* < 0.0001) reduction in fungal cells when co‐cultured with *S. aureus* USA300, Newman and NUI0017, respectively, compared to the mono‐species biofilm (Figure 4B). The LBF *C. albicans* strain NCYC 610 showed a marked reduction in fungal cell numbers in interkingdom biofilms compared to the mono‐species biofilm, with increasing amphotericin B concentration (Figure 4D). Complete eradication of *C. albicans* NCYC 610 was observed in interkingdom biofilms at 4× MBIC amphotericin B, whereas a 1.73‐log reduction was noted in mono‐species biofilms. ATCC 18804 showed increased sensitivity to lower concentrations of amphotericin B when grown as interkingdom biofilms. For instance, at 1× MBIC amphotericin B, *C. albicans* ATCC 18804 biofilms exhibited additional reductions of 1.15‐log, 0.75‐log and 1.96‐log in viable fungal cells when co‐cultured with *S. aureus* USA300 (*p* < 0.0001), Newman (*p* = 0.0012) and NUI0017 (*p* < 0.0001), respectively (Figure 4F). At 4× the MBIC of amphotericin B, the antifungal activity against ATCC 18804 was not significantly different when comparing the mono‐species biofilms with the dual‐species biofilms.

### 3.4 Tolerance of Mono‐ and Dual‐Species Biofilms to Cold Atmospheric Plasma (CAP)

**FIGURE 5:**
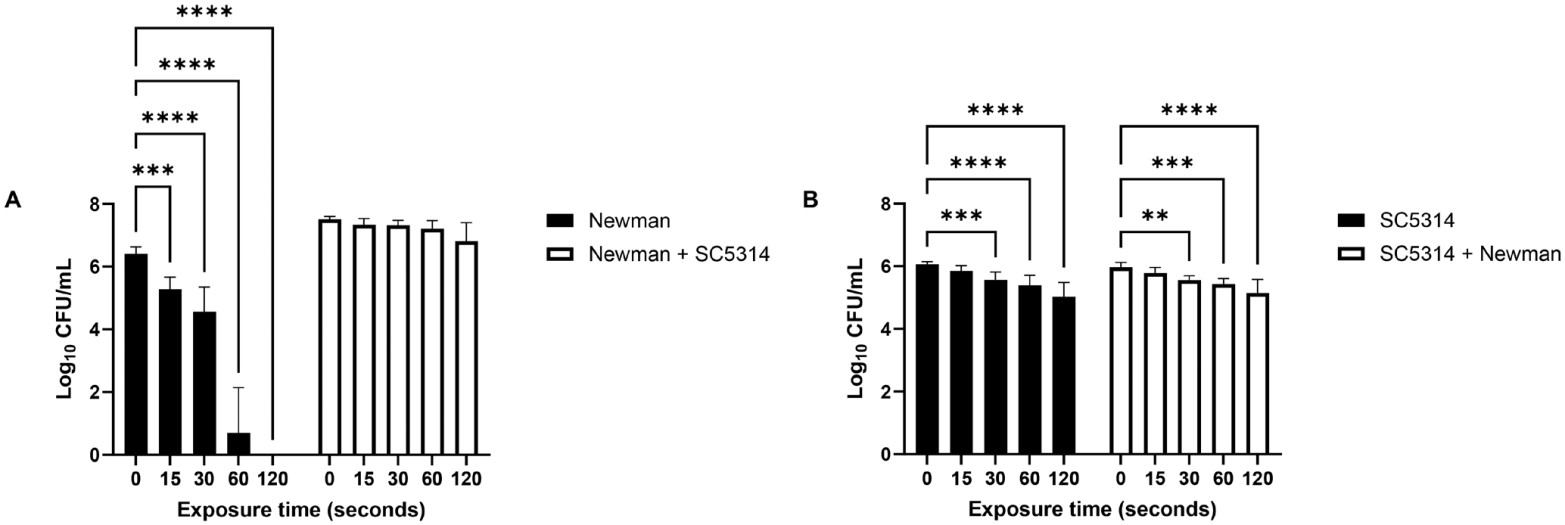
Survival of *S. aureus* Newman and *C. albicans* SC5314 mono and dual‐species biofilms after treatment with the in‐house kHz Jet. Colony recovery of *S. aureus* (A) and *C. albicans* (B) in mono‐ and dual‐species biofilms after CAP treatment using the in‐house kHz jet. Cell recovery was determined using the Miles and Misra method, biofilms were treated in triplicate in 3 separate experiments (*n* = 9). Statistical significance is indicated as follows, **p < 0.01 ***p < 0.0002 and ****p < 0.0001 determined using a two‐way ANOVA, followed by Šídák's multiple comparisons test.

Due to their high biomass (Figure S1), *C. albicans* SC5314 and *S. aureus* Newman were selected for CAP tolerance studies. Exposure to CAP significantly reduced *S. aureus* viability in mono‐species biofilms in‐line in a time‐dependent manner, with a 1.13‐log reduction after 15 s (*p* = 0.0004), a 5.72‐log reduction at 60 s and complete eradication at 120 s (Figure 5A). However, in dual‐species biofilms with SC5314, no significant reduction in *S. aureus* cells was observed after even after 120 s (*p* = 0.0520), highlighting that *C. albicans* confers protections beyond conventional antimicrobials agents but also CAP generated RONS. SC5314 mono‐species biofilms were more tolerant to CAP, with a only a 0.49‐log reduction in fungal cells (*p* = 0.0006) after 30 s (Figure 5B), and gradual decline with longer exposure. We showed that the concentration of H_2_O_2_ increases with exposure time to CAP, ranging from approx. 0.4 mM after 30 s to 0.8 mM after 120 s (Figure S8), which is likely a contributing factor to cell death. Interestingly, there was no significant difference in *C. albicans* counts for any of the CAP exposure times when comparing mono‐species and dual‐species biofilms, suggesting the presence of *S. aureus* does not provide any additional protection to *C. albicans* .

### 3.5 Combined Effect of CAP and Antimicrobials on Dual‐Species Interkingdom Biofilms

**FIGURE 6:**
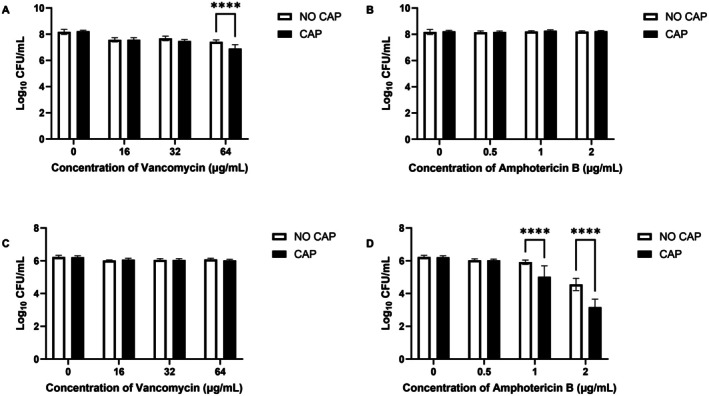
Survival of *S. aureus* Newman and *C. albicans* SC5314 dual‐species biofilms after treatment with the in‐house kHz Jet, followed by antimicrobial exposure. Colony recovery of *S. aureus* (A and B) and *C. albicans* (C and D) in dual‐species biofilms after CAP treatment using the in‐house kHz jet, followed by treatment with either amphotericin B (A and C) or vancomycin (B and D). Cell recovery was determined using the Miles and Misra method; biofilms were treated in quadruplicate in two separate experiments (*n* = 8). Significance is represented by *p < 0.05, **p < 0.01 and ****p < 0.0001, determined by two‐way ANOVA test, followed by Šídák's multiple comparisons test.

CAP pre‐treatment enhanced antimicrobial susceptibility of both organisms in dual‐species biofilms. A 60 s CAP pre‐treatment prior to treatment with with 32 and 64 μg/mL of vancomycin significantly increased *S. aureus* killing activity (Figure 6A). Similarly, when exposed to amphotericin B at concentrations of 1 and 2 μg/mL, CAP pre‐treatment led to a further decrease in the number of yeast cells (Figure 6D). However, no significant additional reduction in the non‐target organism was observed for either antimicrobial following a 60 s CAP pre‐treatment (Figure 6B,C).

**FIGURE 7:**
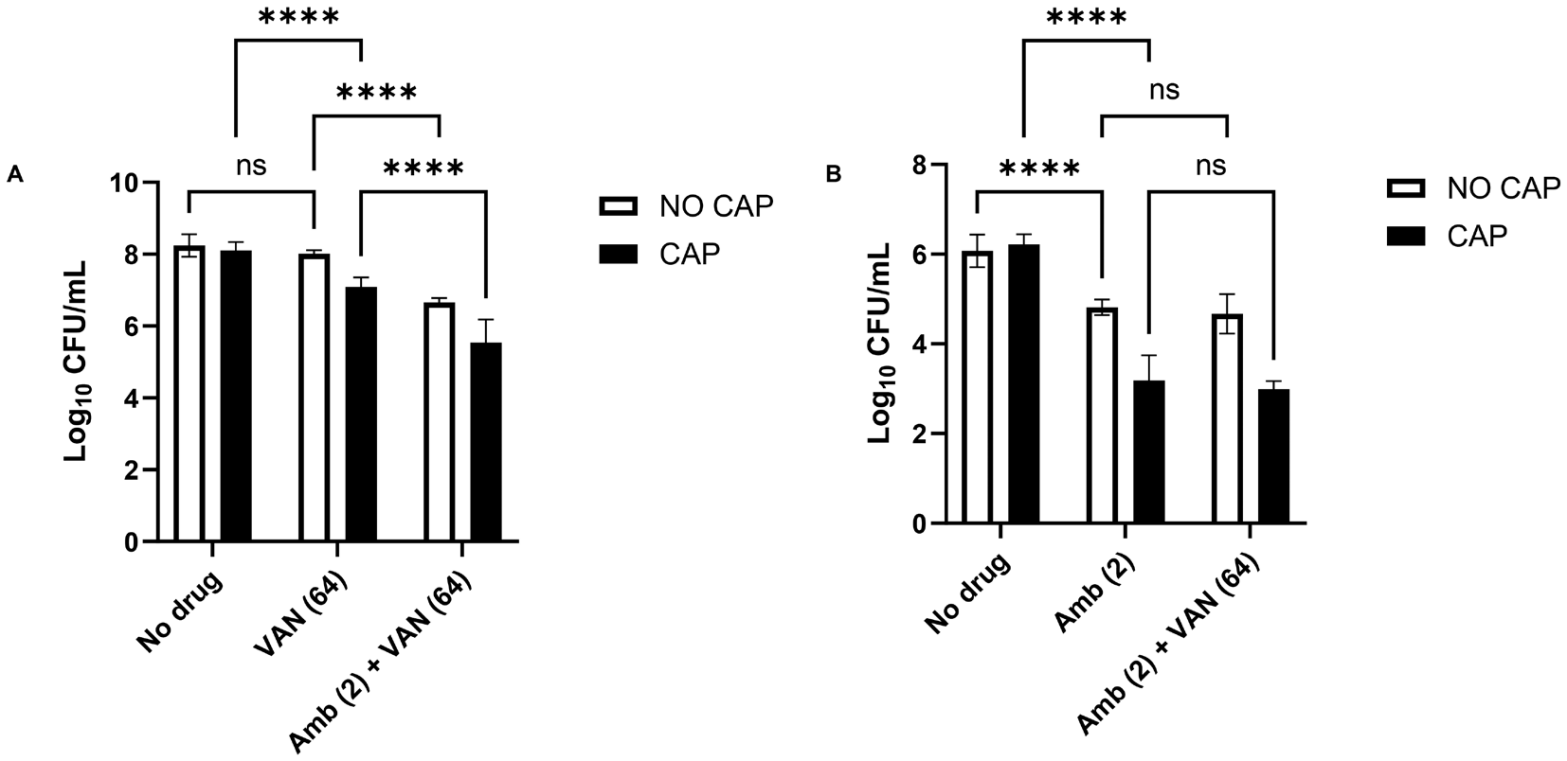
Survival of *S. aureus* Newman and *C. albicans* SC5314 in dual‐species biofilms after treatment with the in‐house kHz Jet, followed by antimicrobial exposure. Colony recovery of *S. aureus* (A) and *C. albicans* (B) in dual‐species biofilms after CAP treatment using the in‐house kHz jet, followed by treatment with amphotericin B and vancomycin. Cell recovery was determined using the Miles and Misra method; biofilms were treated in triplicate in three separate experiments (*n* = 9). Significance is represented by ****p < 0.0001, determined by two‐way ANOVA test, followed by Šídák's multiple comparisons test. Amb (2) = 2 μg/mL of amphotericin B, VAN (64) = 64 μg/mL of vancomycin.

Using a combination of amphotericin B and vancomycin at the highest concentrations tested, following CAP pre‐treatment (Figure 7A), caused the number of *S. aureus* colonies within dual‐species biofilms to be reduced by a total of 2.7‐log. This reduction was significantly greater than the 1.59‐log reduction observed without CAP pre‐treatment (*p* < 0.0001), or the 1.15‐log reduction achieved with vancomycin and CAP alone (*p* < 0.0001). In contrast, the addition of 64 μg/mL of vancomycin did not further reduce the number of *C. albicans* cells in interkingdom biofilms compared to the reduction achieved using 2 μg/mL of amphotericin B combined with CAP pre‐treatment (*p* = 0.56) (Figure 7B).

However, studies on *C. albicans–S. aureus* dual‐species biofilms have almost exclusively focused on the SC5314 strain. To date, no research has confirmed whether this strain is representative of other *C. albicans* strains in interkingdom biofilms. Furthermore, SC5314 has been shown to protect *S. aureus* from antimicrobials, likely by sequestering drugs within the extracellular matrix and extensive hyphal network [8, 9, 10]. This raises the question whether *C. albicans* strains which do not form as robust biofilms can still provide this protection. Regardless of this outcome, a different mechanism should be considered for the treatment of interkingdom biofilms, particularly given the role of the local microenvironment, including nutrient gradients, oxygen diffusion limitations and extracellular matrix (ECM), in shaping biofilm persistence and antimicrobial resistance. CAP has a multi‐mechanism effect on biofilms, including ECM breakdown and cellular inactivation, giving it an advantage over conventional antmicrobial regiments.

## 4 Discussion

Building on previous studies focused on SC5314, we assessed whether strain‐level differences in *C. albicans* influence interkingdom biofilm resilience and susceptibility to CAP. CAP had a substantial effect on *S. aureus* Newman survival in mono‐species biofilms. However, this effect was lost when grown alongside *C. albicans* SC5314, likely due to the significant increase in biomass and ECM produced in dual‐species biofilms. These structural changes are known to limit CAP penetration and reduce reactive species diffusion. This is a common observation in CAP biofilm studies, our group previously reported increased CAP tolerance in *Burkholderia* strains that overproduce ECM [23], and in *Acinetobacter baumannii*, where tolerance increased in more mature, more biomass‐rich biofilms [25]. Rather than acting as non‐discriminatory biofilm disruptor, CAP could be selectively disrupting the infection microenvironment, transiently weakening ECM integrity and redox balance to imporve antimicrobial penetration.

*C. albicans* is more tolerant to hydrogen peroxide in its hyphae form, and sub‐lethal oxidative conditions can induce this filamentation transition [42]. It is therefore likely that after short CAP exposures, only limited RONS, including hydrogen peroxide, react with *C. albicans* and longer exposure times are required to overcome the tolerance of the hyphae already present within the biofilms. A previous study looked into the effects of CAP on dual‐species biofilms of *C. albicans* and *S. aureus* and did not find any significant increase in survival of bacterial cells compared to mono‐species biofilms [43]. However, their plasma source (kINPen) had a much lesser effect on the *S. aureus* mono‐species biofilm compared to the in‐house kHz jet used in this study. In another triadic biofilm model with *C. albicans* , *P. aeruginosa* and *S. aureus* , CAP tolerance did not increase in co‐culture, although *S. aureus* outcompeted other organisms post‐treatment [44], highlighting the potential role of competitive metabolic interactions in biofilm resilience.

The effects of CAP were further enhanced by the addition of vancomycin or amphotericin B. Pre‐treatment with CAP, followed by either antimicrobial led to significantly greater reductions in viable cell numbers compared to antimicrobials alone. The triple combination of CAP, vancomycin and amphotericin B yielded the greatest effect on *S. aureus* , likely due to disruption of hyphal networks due to CAP‐generated hydrogen peroxide, leading to a destabilisation of the biofilm matrix and increased drug penetration. Furthermore, a synergistic effect between CAP and antimicrobials has been previously reported in mono‐species *Pseudomonas aeruginosa* biofilms, which was thought to be partly attributable to RONS‐induced of oxidative stress [26]. While combinations of CAP and bacteriophages were also shown to have greater antimicrobial effects against *Proteus mirabilis* biofilms compared to using either treatment alone [45]. While dual treatment with vancomycin and amphotericin B did further reduce viable cell counts, full eradication of either species was not achieved, underscoring the persistent challenge of polymicrobial biofilms. Interestingly, combinational treatment with the highest concentrations of amphotericin B and vancomycin in the absence of CAP, had minimal additional effects on cell reduction in interkingdom biofilms. However, Investigating a wider range of concentrations could yield valuable insights into how these two organisms interact and adapt under antimicrobial stress.

We also show that interkingdom biofilm architecture and cell composition are primarily governed by the *C. albicans* strain. HBF strains such as SC5314 formed dense hyphal matrices that promoted increased *S. aureus* biomass, regardless of the bacterial strain. In contrast, LBF strains like NCYC 610 produced less ECM and were more influenced by the *S. aureus* strain present. Despite this variability, all *C. albicans* strains conferred increased vancomycin tolerance to *S. aureus* , although the degree varied.

Protection by SC5314 and ATCC 18804 was likely driven by multple microenvironmental factors, including the dense biofilm matrix sequestering vancomycin, oxygen and nutrient‐limited niches favouring bacterial persistence [46] and a complex hyphal network providing physical refuges where *S. aureus* can evade exposure to lethal drug concentrations [9]. NCYC 610 showed no signs of hyphae formation and therefore lacked the dense filamentous meshwork seen in the other two strains. We initially hypothesised that co‐culture with *S. aureus* might induce hypha formation in NCYC 610, but, this was not suppported by microscopy analysis (Figure S9). The modest protection observed may instead be mediated by β‐1,3 glucans alone [47], which could partially sequester vancomycin but would be less effective than the combined barrier effects seen in HBF and IBF strains.

In contrast to this protection, *C. albicans* itself became more susceptible to amphotericin B when grown in co‐culture. We propose this is due to the combined metabolic stress of antifungal exposure and nutrient competition with *S. aureus* . Membrane disruption under these conditions likely compromises *C. albicans* survival, while *S. aureus* continues to exploit residual matrix components for its own persistence. However, it should also be considered that studying these biofilms at 30°C may yield a different architecture/metabolism and potentially alter each organism's sensitivity to both antimicrobials and CAP.

Taken together, these findings suggest that *C. albicans* , regardless of strain, protects *S. aureus* within interkingdom biofilms, including against CAP. However, CAP acts as a powerful anti‐biofilm adjunct, disrupting biofilm architecture and enabling antimicrobial agents to penetrate more effectively. While complete eradication remains elusive, this combinatorial approach warrants further exploration for clinically intractable infections.

## 5 Conclusions

Interkingdom biofilms between *C. albicans* and *S. aureus* exhibit significant variability depending on the specific strains involved. However, the protection against vancomycin provided by *C. albicans* during interactions with *S. aureus* appears to be a universal trait, with the extent of protection correlating to the biofilm‐forming ability of the *C. albicans* strain. The use of CAP enhances the antimicrobial activity of both vancomycin and amphotericin B against interkingdom biofilms and the immuno‐stimulatory effects of CAP makes it a promising technology to help in the resolution of a variety of complex infections.

## Funding

This work was supported by the Medical Research Council (MC_PC_20019 and MR/X014010/1), Public Health Agency (STL/5350/17) and Department for the Economy.

## Ethics Statement

No animals, patient samples or human tissue were used in this research. No patient data was collected or analysed. All research involved in vitro laboratory experiments.

## Conflicts of Interest

The authors declare no conflicts of interest.

## Supporting information


**Data S1:** apm70119‐sup‐0001‐supinfo.docx.

## Data Availability

The data that support the findings of this study are available from the corresponding author upon reasonable request.
