# Supplementary material for: Understanding Heterogeneity and Tolerance of Dual Candida albicans–Staphylococcus aureus Biofilms to Cold Atmospheric Plasma and Antimicrobial Combinations
Source: APMIS. 2025 Dec 29;133(12):e70119. doi: 10.1111/apm.70119 (PMC12746060; doi:10.1111/apm.70119)
Supplement: Supplementary file 1 — Data S1: apm70119‐sup‐0001‐supinfo.docx. [file APM-133-0-s001.docx]

**Supplementary Figures**


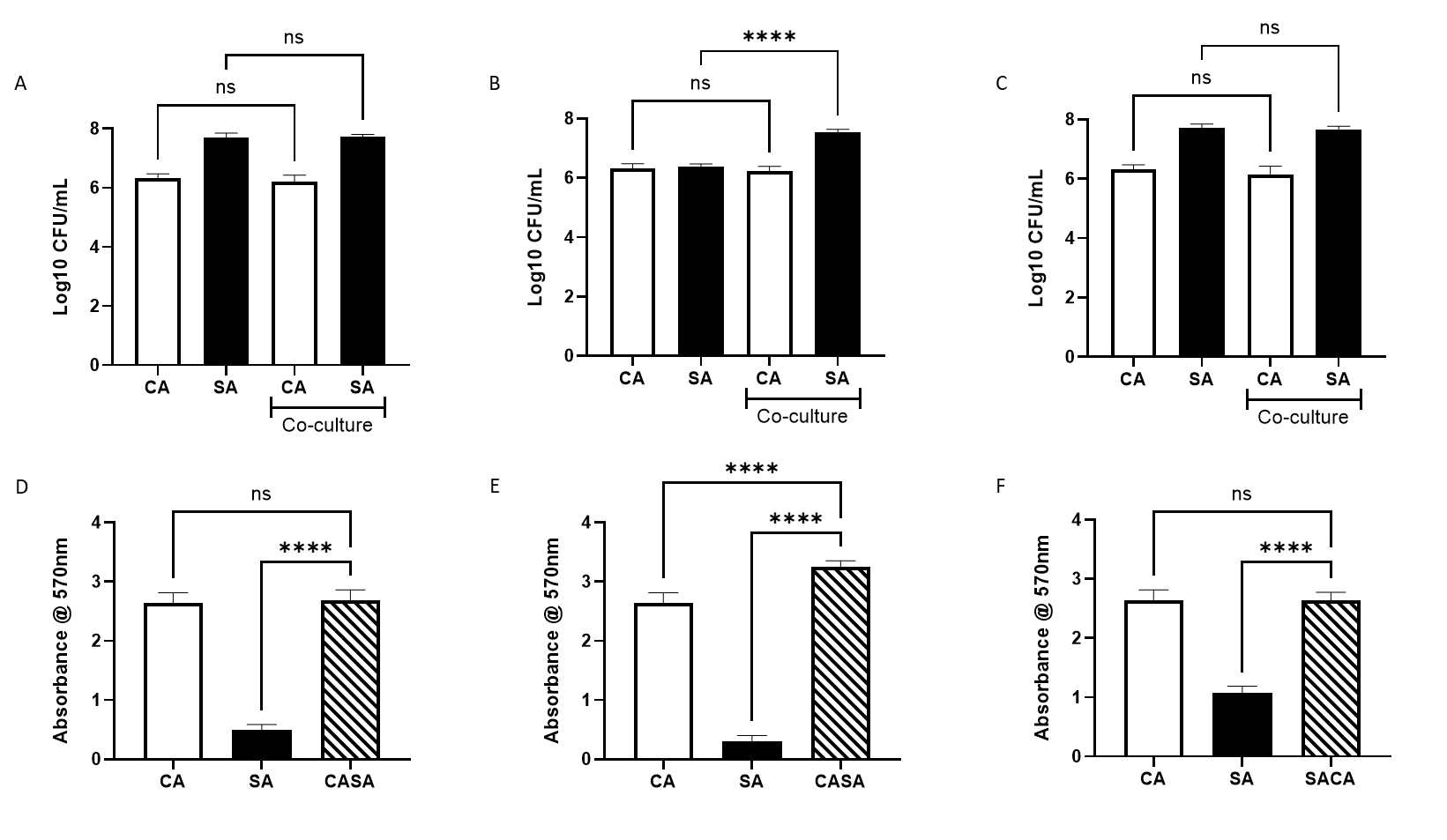


**Supplementary Fig. 1: CFU/mL and total biomass of Interkingdom biofilms between *C. albicans* SC5314 and *S. aureus* strains.** Biofilms between *C. albicans* SC5314 and *S. aureus* USA300 (A and D), Newman (B and E) or NUI0017 (C and F) were grown for 24 hours in RPMI before CFU/mL was calculated using the miles and mirsa plate count method (A, B and C) and total biomass using the crystal violet assay (D, E and F). CA = *C. albicans,* SA = *S. aureus,* CASA *= C. albicans + S. aureus* biofilm. Significance is represented by ‘***’ P *< 0.0002 and ‘****’ P < 0.0001* determined by one-way ANOVA test followed by Šídák's multiple comparisons test.


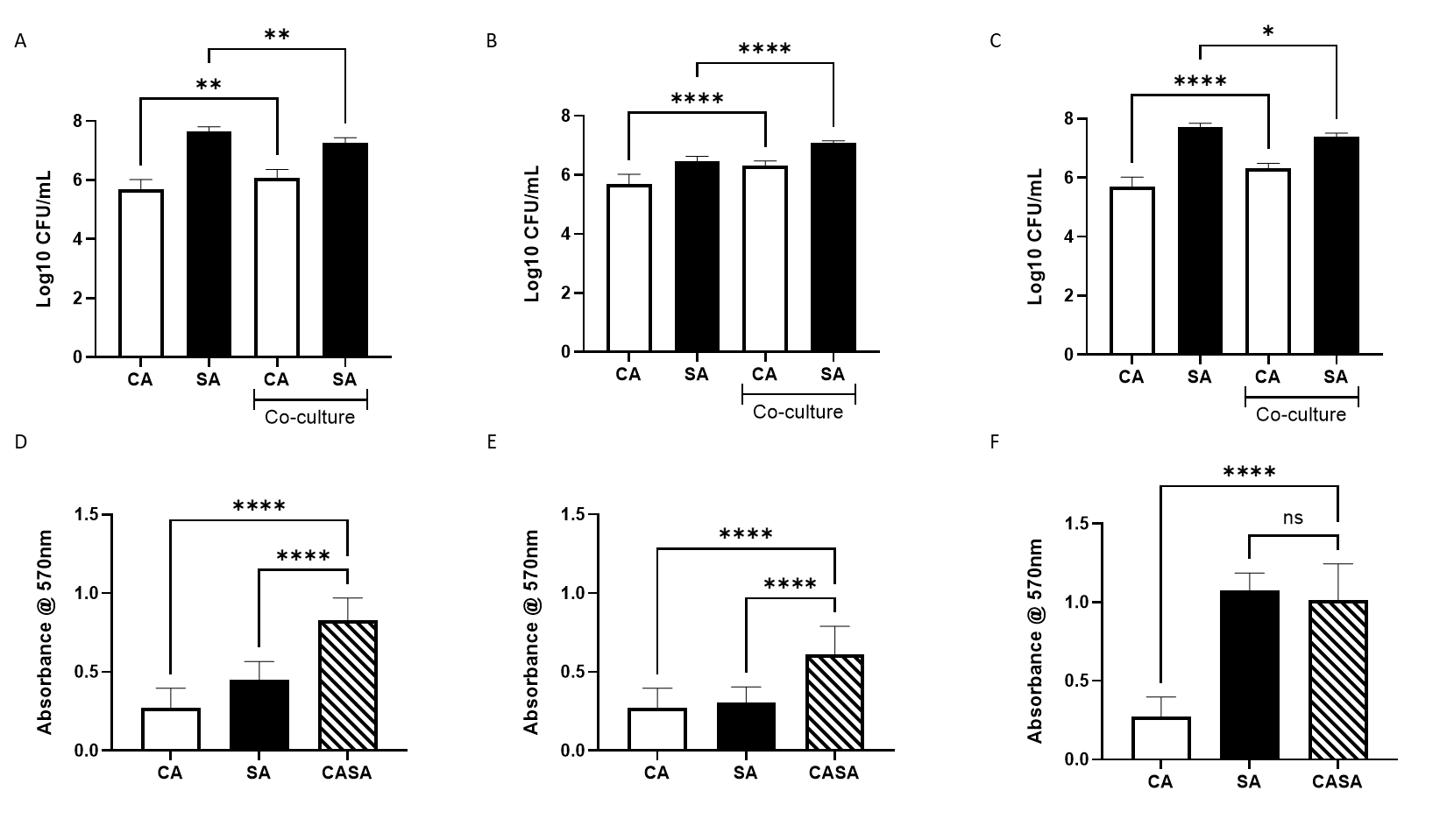


**Supplementary Fig. 2: CFU/mL and total biomass of Interkingdom biofilms between *C. albicans* NCYC 610 and *S. aureus* strains**. Biofilms between *C. albicans* NCYC 610 and *S. aureus* USA300 (A and D), Newman (B and E) or NUI0017 (C and F) were grown for 24 hours in RPMI before CFU/mL was calculated using the miles and mirsa plate count method (A, B and C) and total biomass using the crystal violet assay (D, E and F). CA = *C. albicans,* SA = *S. aureus,* CASA *= C. albicans + S. aureus* biofilm. Significance is represented by ‘**’ P < 0.01 and ‘****’ P < 0.0001 determined by one-way ANOVA test followed by Šídák's multiple comparisons test.


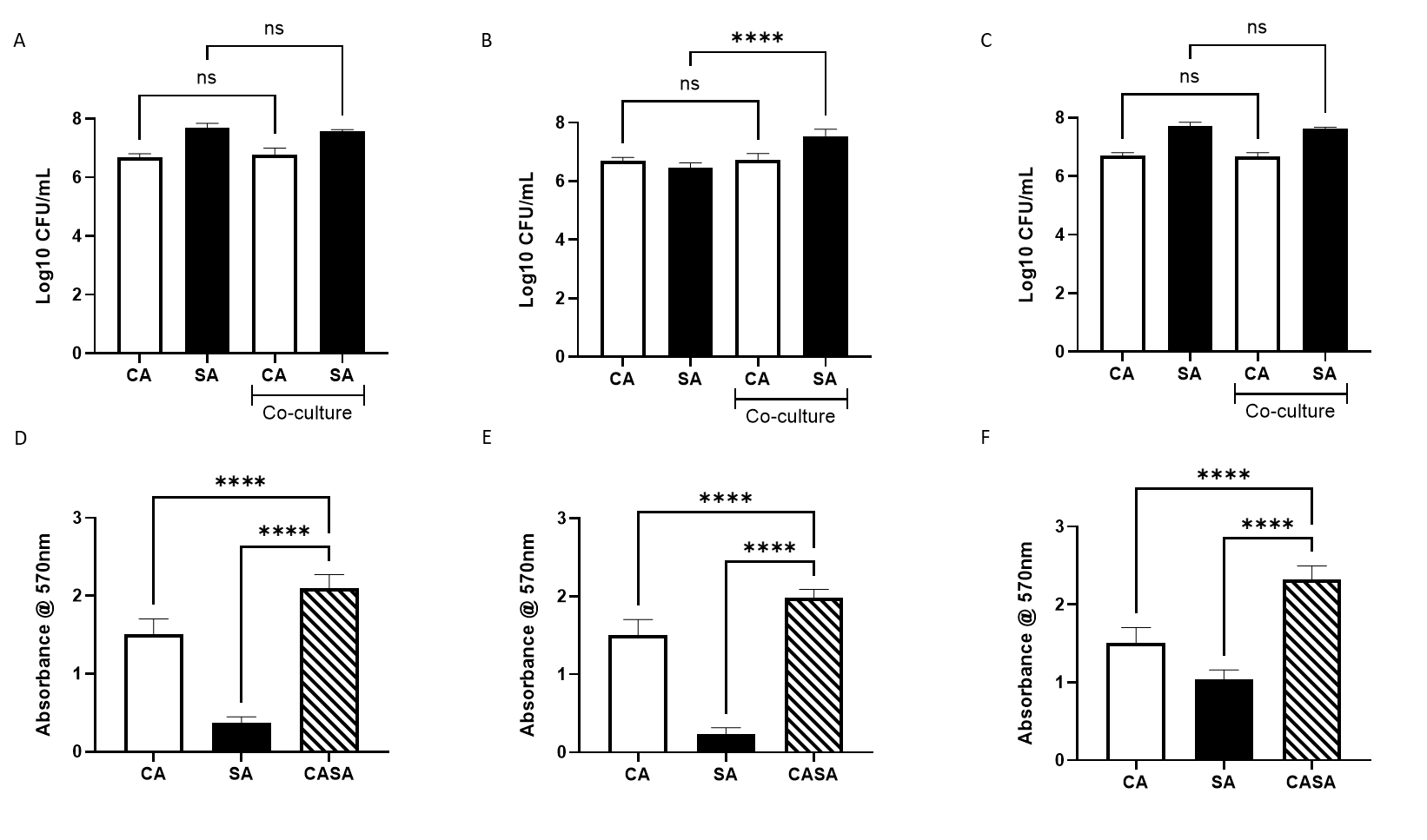


**Supplementary Fig. 3: CFU/mL and total biomass of Interkingdom biofilms between *C. albicans* ATCC 18804 and *S. aureus* strains.** Biofilms between *C. albicans* ATCC 18804 and *S. aureus* USA300 (A and D), Newman (B and E) or NUI0017 (C and F) were grown for 24 hours in RPMI before CFU/mL was calculated using the miles and mirsa plate count method (A, B and C) and total biomass using the crystal violet assay (D, E and F). CA = *C. albicans,* SA = *S. aureus,* CASA *= C. albicans + S. aureus* biofilm. Significance is represented by ‘***’ P < 0.0002 and ‘****’ P < 0.0001 determined by one-way ANOVA test followed by Šídák's multiple comparisons test.


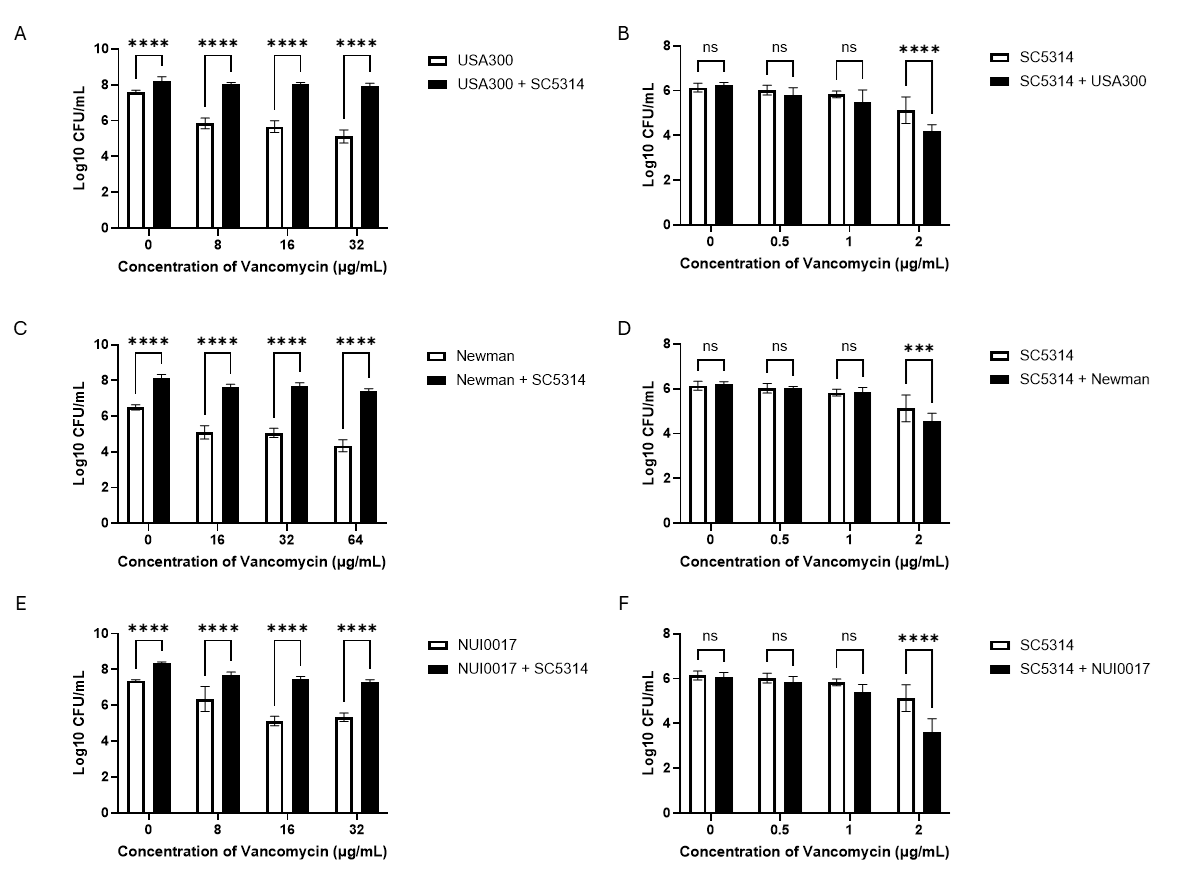


**Supplementary Fig. 4: Vancomycin and Amphotericin B tolerance of Interkingdom biofilms between *C. albicans* SC5314 and *S. aureus*.** *S. aureus* strains BAA 1717 (A and B), NCTC 10833 (C and D) and NUI0017 (E and F) were grown in the presence of *C. albicans* SC5314 for 24 hours. Biofilms were then subjected to varying vancomycin concentrations at equal to/or above the MBIC for a further 24 hours prior to estimation of cfu/mL of either *S. aureus* or *C. albicans* cells using the miles and misra method. N = 9. Significance is represented by ‘**’ P < 0.01 ‘***’ P < 0.0002 and ‘****’ P < 0.0001 determined by two-way ANOVA test followed by Šídák's multiple comparisons test.


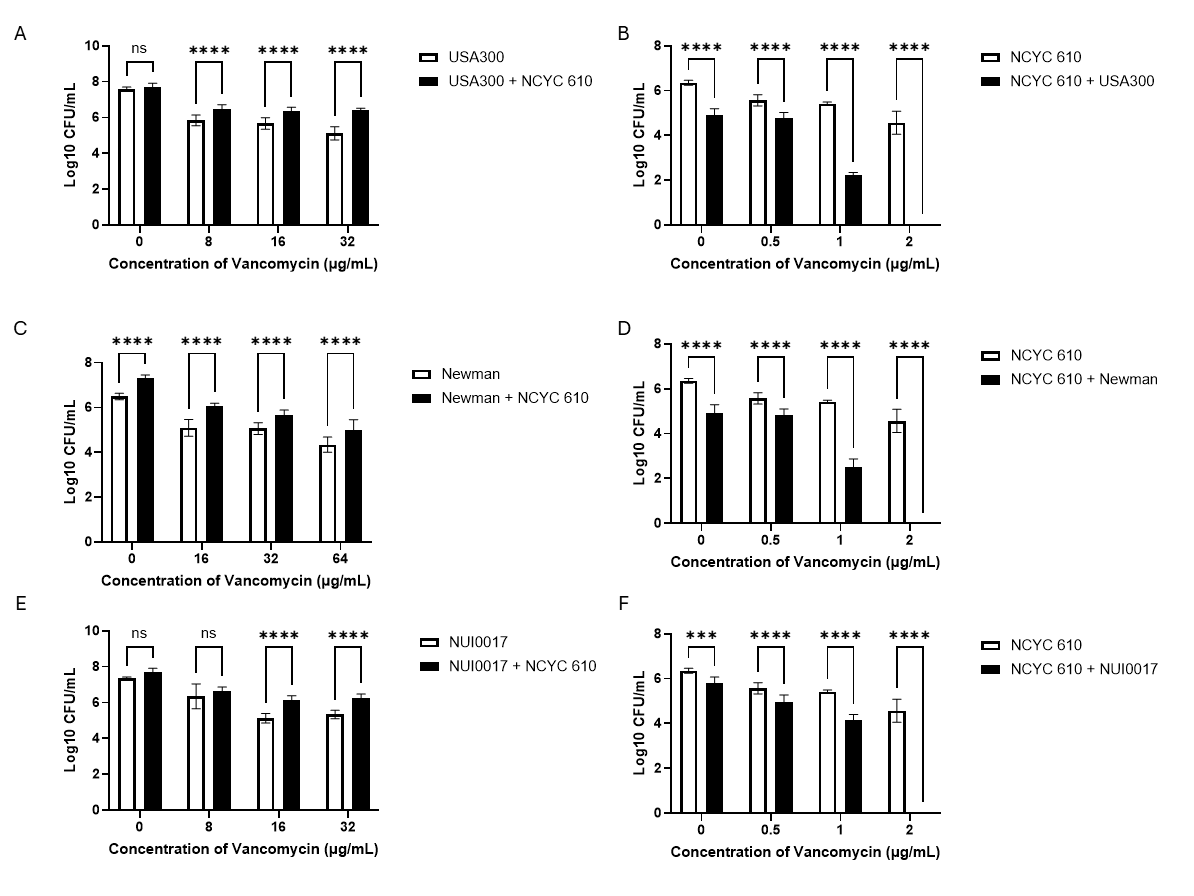


**Supplementary Fig. 5: Vancomycin and Amphotericin B tolerance of Interkingdom biofilms between *C. albicans* NCYC 610 and *S. aureus*.** *S. aureus* strains BAA 1717 (A and B), NCTC 10833 (C and D) and NUI0017 (E and F) were grown in the presence of *C. albicans* NCYC 610 for 24 hours. Biofilms were then subjected to varying vancomycin concentrations at equal to/or above the MBIC for a further 24 hours prior to estimation of cfu/mL of either *S. aureus* or *C. albicans* cells using the miles and misra method. N = 9. Significance is represented by ‘**’ P < 0.01 ‘***’ P < 0.0002 and ‘****’ P < 0.0001 determined by two-way ANOVA test followed by Šídák's multiple comparisons test.


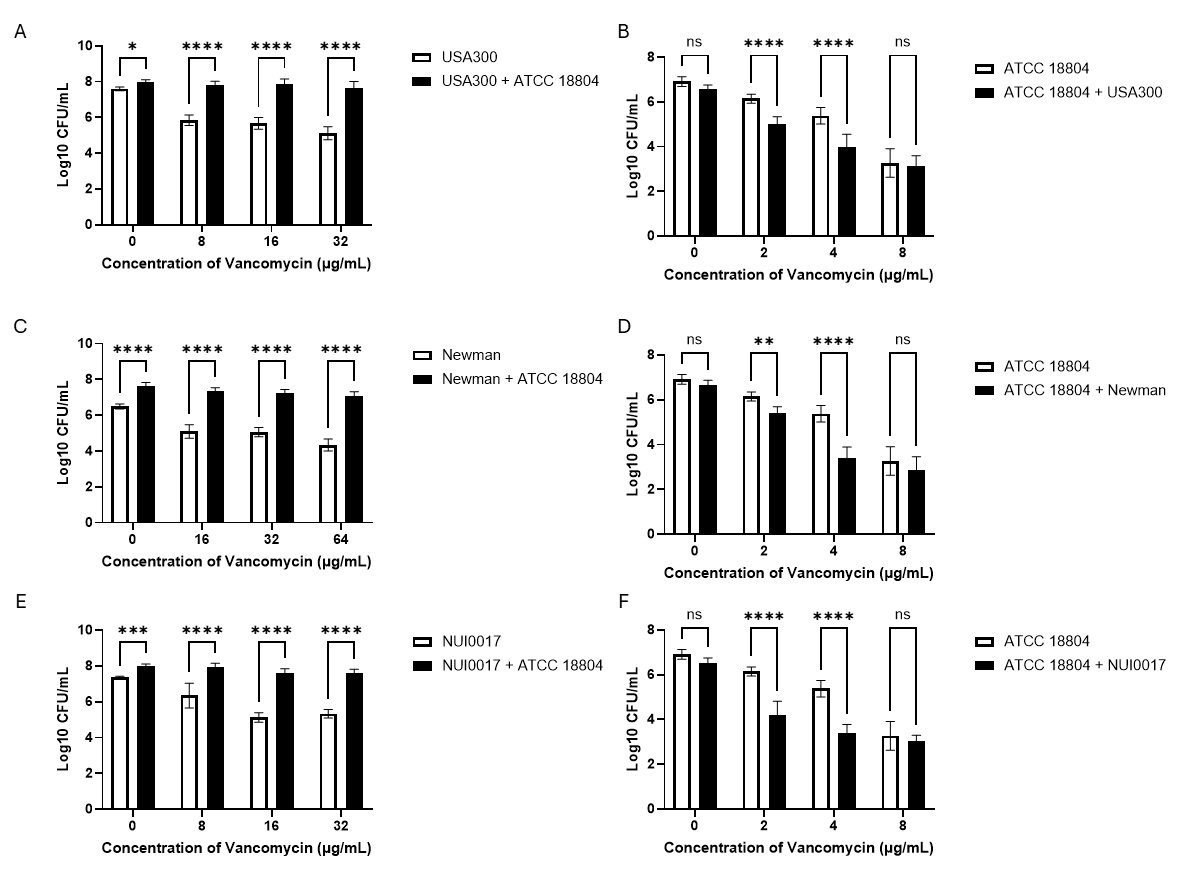


**Supplementary Fig. 6: Vancomycin and Amphotericin B tolerance of Interkingdom biofilms between *C. albicans* ATCC 18804 and *S. aureus*.** *S. aureus* strains BAA 1717 (A and B), NCTC 10833 (C and D) and NUI0017 (E and F) were grown in the presence of *C. albicans* ATCC 18804 for 24 hours. Biofilms were then subjected to varying vancomycin concentrations at equal to/or above the MBIC for a further 24 hours prior to estimation of cfu/mL of either *S. aureus* or *C. albicans* cells using the miles and misra method. N = 9. Significance is represented by ‘*’ P < 0.05, ‘**’ P < 0.01 ‘***’ P < 0.0002 and ‘****’ P < 0.0001 determined by two-way ANOVA test followed by Šídák's multiple comparisons test.


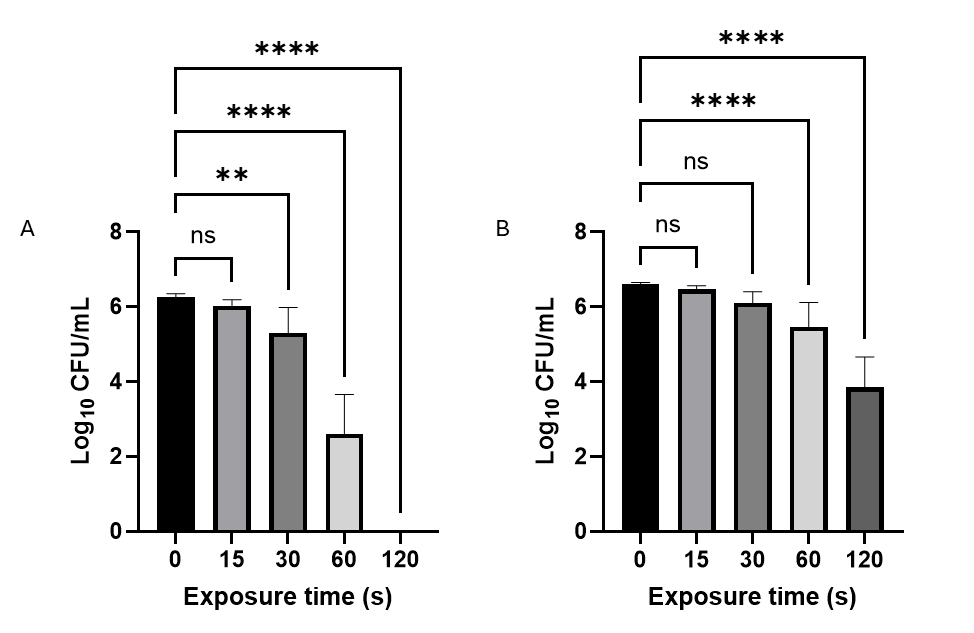


**Supplementary Fig. 7: Survival of *C. albicans* NCYC 610 and ATCC 18804 after treatment with the in-house kHz jet.** Colony recovery of *C. albicans* after NCYC 610 (A) and ATCC 18804 (B) biofilm exposure to the in-house kHz Jet was determined using the Miles and Misra method. Biofilms were treated in triplicate in 3 separate experiments (n = 9). Significance is represented by ‘**’ P < 0.01 and ‘****’ P <0.0001 compared to the 0 second data, determined by the one-way ANOVA test followed by Dunnett’s multiple comparison test.

**
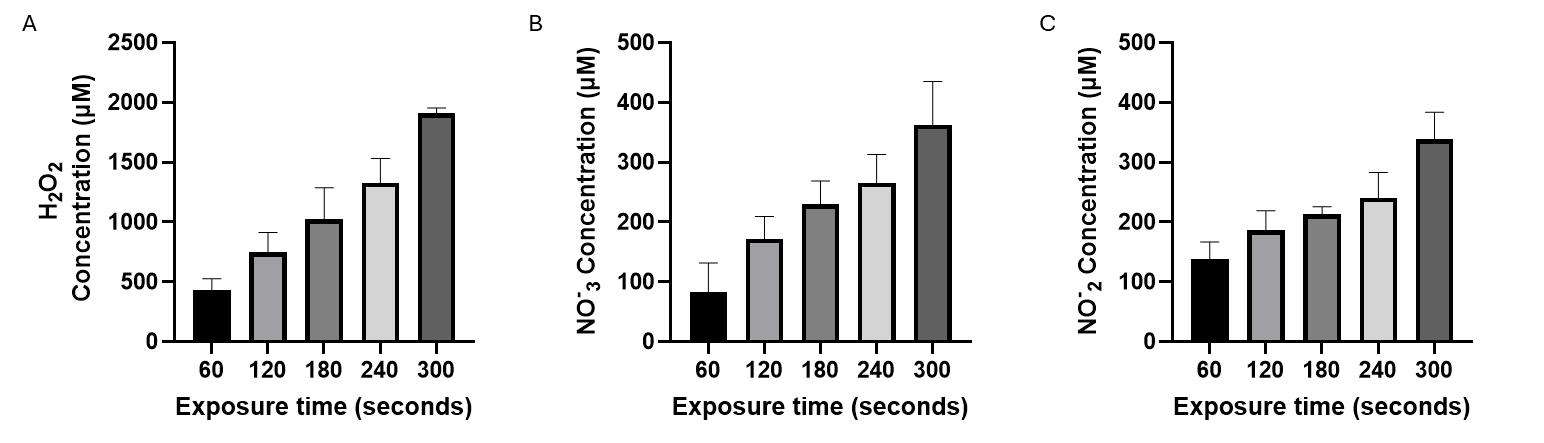
**

**Supplementary Fig. 8: Reactive oxygen and nitrogen species produced in water after CAP exposure.** The concentration of H_2_O_2_ (A), NO^-^_3_ (B) and NO^-^_2_ (C) in CAP-treated water was determined using different colorimetric assays along with standard curves.

**
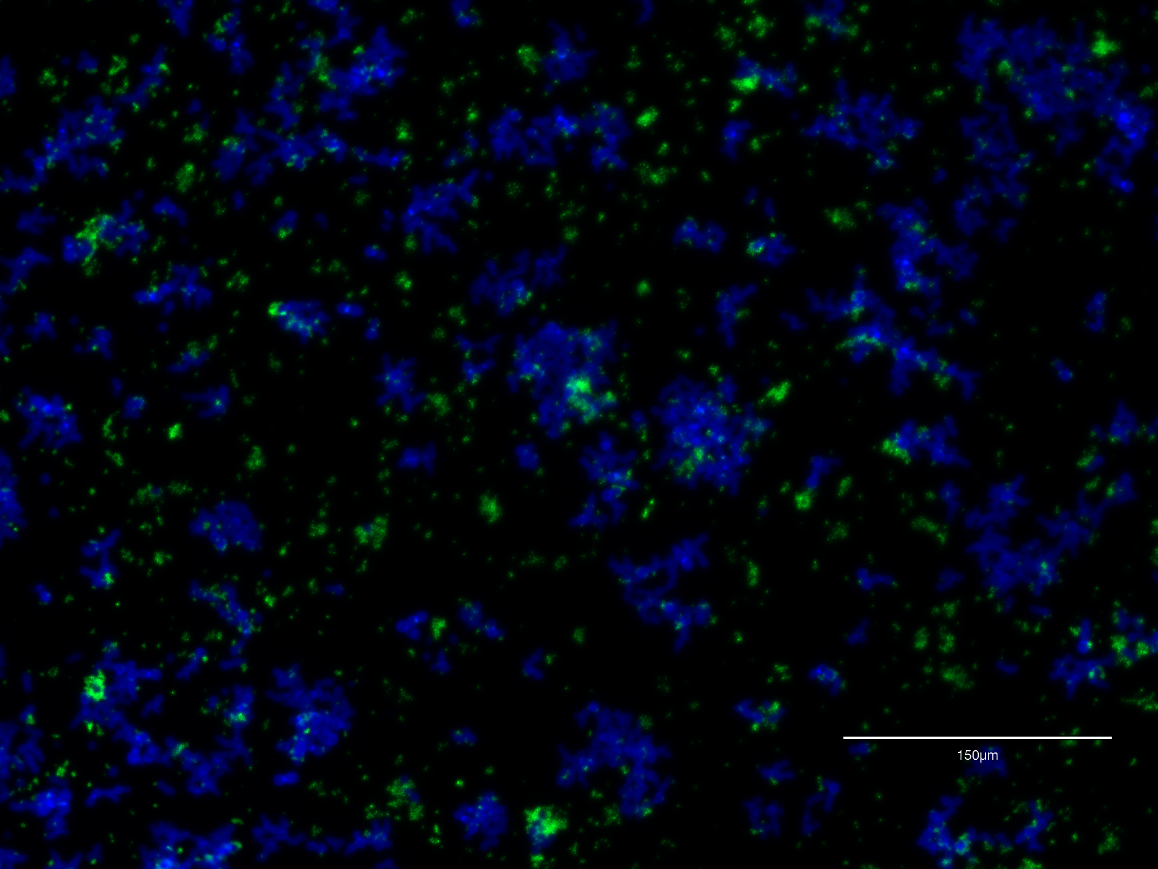
**

**Supplementary Fig. 9. NCYC 610 biofilm grown alongside *S. aureus* GFP-USA300.** NCYC 610 was stained with calcofluor white (blue) and USA300 AH1726 producing GFP (green).
